# Contribution of next generation sequencing in pediatric practice in Lebanon. A Study on 213 cases

**DOI:** 10.1002/mgg3.480

**Published:** 2018-10-07

**Authors:** Pratibha Nair, Sandra Sabbagh, Hicham Mansour, Ali Fawaz, Ghassan Hmaimess, Peter Noun, Rawane Dagher, Hala Megarbane, Sayeeda Hana, Saada Alame, Maher Lamaa, Dana Hasbini, Roula Farah, Mariam Rajab, Samantha Stora, Oulfat El‐Tourjuman, Pauline Abou Jaoude, Gihad Chalouhi, Rony Sayad, Anne‐Celine Gillart, Mahmoud Al‐Ali, Valerie Delague, Stephany El‐Hayek, André Mégarbané

**Affiliations:** ^1^ Centre for Arab Genomic Studies Dubai UAE; ^2^ Pediatric Department Hôtel Dieu de France Hospital Beirut Lebanon; ^3^ Pediatric Department, Saint George Hospital Balamand University Beirut Lebanon; ^4^ Neuropediatrics Department Lebanese University, Clemenceau Medical center Beirut Lebanon; ^5^ Pediatric Department, Notre Dame de Secours University Hospital Byblos Lebanon; ^6^ Dermatology Department, Saint George Hospital Balamand University Beirut Lebanon; ^7^ Pediatric Department El‐Rassoul Hospital Beirut Lebanon; ^8^ Pediatric Neurology Department Rafic Hariri University Hospital Beirut Lebanon; ^9^ Pediatric Department Makassed Hospital Beirut Lebanon; ^10^ Institut Jérôme Lejeune Paris France; ^11^ Simechol Ecole d’enseignement et de Perfectionnement en Echographie Sur Simulateurs Paris France; ^12^ Pediatric Department Abou Jaoude Hospital Beirut Lebanon; ^13^ Aix Marseille Univ Inserm, MMG, U 1251 Marseille France

**Keywords:** consanguinity, gene, panel, variants, whole exome sequencing

## Abstract

**Background:**

According to the Catalogue of Transmission Genetics in Arabs, less than half of diseases reported in Lebanese patients are mapped. In the recent years, Next Generation Sequencing (NGS) techniques have significantly improved clinical diagnosis, compared to traditional sequencing methods.

**Methods:**

A total of 213 analyses by NGS (167 by whole exome sequencing (WES) and 46 by multigene panels tests) were performed on pediatric patients across different regions of Lebanon over a period of two years (December 2015–December 2017).

**Results:**

Neurological disorders were the most frequent referral demand for both WES and gene panels (122/213). Pathogenic, likely pathogenic, or variants of unknown significance were identified in 69.5% of the WES and panel patients combined. Over half of the patients with such variants had an autosomal recessive disorder. A definite molecular diagnosis (pathogenic or likely pathogenic variants) was achieved in 34.1% and 47.8% of the patients studied by WES and the multigene panels, respectively. Thirty‐three novel variants were found in the cases that were molecularly solved; 26 of these being identified by WES and seven by the multigene panels. In three consanguineous families, autosomal recessive inheritance of genes previously reported as showing dominant inheritance patterns were found. Biallelism was found in six cases, digenism in four cases, and one case was trigenic.

**Conclusion:**

Our study thus suggests that NGS tools are valuable for an improved clinical diagnosis, and highlights that the increased adoption of such techniques will significantly further improve our understanding of the genetic basis of inherited diseases in Lebanon.

## 1 INTRODUCTION

The most recent review of genetic disorders in Lebanon reports a total of 378 diseases diagnosed in Lebanese individuals, most of which are not accompanied by any molecular analysis (Nakouzi, Kreidieh, & Yazbek, 2015). According to the Catalogue of Transmission Genetics in Arabs (CAGS, 2018), less than half of diseases reported in Lebanese patients are mapped. In the recent years, with the advent of newer molecular techniques in Lebanon, this has begun to change. Next Generation Sequencing (NGS) techniques have significantly improved clinical diagnosis, compared to traditional sequencing methods (LaDuca et al., 2017; Neveling et al., 2013). In fact, with the adoption of techniques such as array‐CGH, gene panels, and whole exome/genome sequencing in Lebanon, not only has the identification of the origin of various disorders been enhanced, but such techniques have also helped more accurately confirm or correct previous diagnoses (Megarbane, 2018). Still, patients in Lebanon often tend to opt out of the recommended genetic testing, as such tests are not covered by insurance and thus have to be personally financed.

In this report, we present the results of a study which included a total of 213 analyses by NGS (167 by whole exome sequencing (WES) and 46 by multigene panels tests) performed on pediatric patients, over a period of two years (December 2015–December 2017). Our analyses identified positive results in 69.5% of the patients who underwent either WES or panel testing. Our study thus suggests that these tools are valuable for an improved clinical diagnosis, and highlights that the increased adoption of such techniques will significantly further improve our understanding of the genetic basis of inherited diseases in Lebanon.

## 2 MATERIALS AND METHODS

### 2.1 Ethical compliance

This study is conformed to the tenets of the Declaration of Helsinki, and was supervised and approved by an international ethical committee.

### 2.2 Patients

The patients included in this series were referred for genetic counselling by their treating physician, mostly pediatricians and neurologists. Patients were below the age of 16, came from different regions of Lebanon, and were seen over a period of two years (December 2015–December 2017). Informed consent for genetic analysis was obtained from the patients’ parents, in compliance with national ethics regulation. For the patients who underwent WES analysis, the possibility to reveal incidental findings that are not necessarily related to the reason for ordering the sequencing but could still be of medical importance, was also discussed, with the option to decline receiving such findings. Incidental findings were reported in accordance with the ACMG recommendations, and taken from the list of 59 actionable genes (Kalia et al., 2017).

For WES, only the index patients were sequenced. Approximately 37 Mb (214,405 exons) of the Consensus Coding Sequences (CCS) were enriched from fragmented genomic DNA by more than 340,000 probes designed against the human genome (Nextera Rapid Capture Exome, Illumina) and the generated library sequenced on an Illumina NextSeq or HiSeq 4,000 platform (Illumina) to an average coverage depth 70–100X. An end to end bioinformatics pipelines including base calling, primary filtering of low quality reads and probable artefacts, and annotation of variants was applied.

For panel tests, different clinically themed multigene panels were offered: neurological disorders including seizures and neuromuscular disorders, inborn errors of metabolism, primary immunodeficiency and fever of unknown origin, oncology, renal diseases, dermatological disorders, and cardiac malformations. Genomic DNA obtained from the submitted sample was enriched for targeted regions using a hybridization‐based protocol, and sequenced. All targeted regions were sequenced with ≥50x depth. In case of a normal result, a search for deletion/duplication was performed as well using Multiplex Ligation‐dependent Probe Amplification (MLPA) technique.

### 2.3 Mutational analysis

The in silico bioinformatic tool MutationTaster (https://www.mutationtaster.org/) was used to predict the effect of the identified variant. Variant novelty was assessed based on its absence from public variant repositories including GnomAD, and 1000G, as well as an in‐house database housing over 715 exomes belonging to Arab individuals from the Arabian Peninsula. To assess conservation of the point of insertion and subsequent residues, multiple protein sequence alignment across multiple species was obtained from Homologene (https://www.ncbi.nlm.nih.gov/homologene).

According to the ACMG recommendations (Richards et al., 2015), variants were classified as: Class 1: pathogenic variant; class 2: likely pathogenic variant; class 3: variant of unknown significance; class 4: probably non pathogenic variant; class 5: benign/normal variant. A positive result was considered when variants of class 1 or 2 were identified and when a class 3 variant was found, as these are potentially positive. The identification of class 1 or 2 variants was considered a definite molecular diagnosis.

## 3 RESULTS

A total of 213 pediatric patients were included in this series. A WES was performed for 167 patients and for 46 of them, a multigene panel was used.

In 108 of the WES patients, a positive result was considered and 123 variants were found: 61 of class 1–2 of which 26 novel ones (Table 1); and 62 of class 3. Fifty‐eight patients were homozygous, three compound heterozygous, 12 had two genes possibly involved in their pathology, and 34 patients were heterozygous. In one patient, class 1 and class 2 variants in three genes (*THOC6* (OMIM 615,403)*, PTCH2* (OMIM 603,673) and* EDAR* (OMIM 604,095)) were found to be at the origin of their clinical features.

**Table 1:** Variants identified by WES in our patients

Gene	Transcript	cDNA	Protein	Novelty	Primary manifestation	Diagnosis post WES
Heterozygous variants						
*KMT2A*	NM_001197104.1	c.2627_2630del	p.Arg876Thrfs	Novel	DD; ID; Short stature (Neurological)	Wiedeman Steiner disease
*RYR1*	NM_000540.2	c.8758C>T	p.Arg2920*	Novel	DD, MD; joint contratures; elevated CK; slightly increased lactic acid (Neurological)	Central core disease
*LDB3*	*NM_001171610.1*	*c.694G>A*	*p.D232N*	*rs121908338*		
*CDHR1*	NM_033100.3	c.420T>A	p.Tyr140*	Novel	Cone‐rod dystrophy; visual impairment (Ophthalmological)	Retinitis pigmentosa 65
*GATA2*	NM_032638.4	c.1032_1036dup	p.Gly346Glufs	Novel	Hearing impairment; motor delay; lower limb edema (Immune/hematology)	Immune deficiency
*SMARCA4*	NM_001128849.1	c.3506A	p.Asp1169Gly	Novel	DD; coarse face; encephalocele; omphalocele; bifid uvula; sub mucous cleft palate; ataxia; muscular hypotonia (Multiple system disease)	Coffin‐Siris type 4
*CACNA1A*	NM_023035.2	c.997A>G	p.Asn333Asp	Novel	DD; ID; Hypotonia; seizures (Neurological)	Infantile epileptic encephalopathy type 42
*NF1*	NM_001042492.2	c.1019_1020del	p.Ser340Cysfs	Novel	Café‐au‐lait spots (Dermatological)	Neurofibromatosis
*PTCH2^§^*	NM_003738.4	c.528del	p.Met176Ilefs	Novel	hydrocephalus; DD; ID; abnormal gait; carse face, malformation of heart and great vessels; short stature; undescended testis; micropenis (Multiple system)	
*RET*	NM_020975.4	c.2735G>A	p.R912Q	rs78347871		
*PKHD1*	*NM_138694.3*	*c*.*5086 T>G*	*p.S1696A*	*Novel*	Spina bifida; Polycystic kidney; hepatic fibrosis (Renal)	Polycystic kidney disease
*COL7A1*	NM_000094.3	c.6082G>A	p.Gly2028Arg	rs762162799	Epidermolysis bullosa (Dermatological)	Epidermolysis bullosa, Bart type
*FGA*	NM_000508.3	c.2587del	p.V864X	rs773678959	Thromboembolic cerebral accident; short extremities; seizures (Immune/hematology)	Congenital dysfibrinogenemia
*KANSL1*	NM_015443.3	c.808_809del	p.L270Vfs	rs551541795	DD; ID; muscular hypotonia; ptosis; short stature; hyperlaxity (Multiple systems)	Koolen‐De Vries syndrome
*COMP*	NM_000095.2	c.2156G>A p	p.Gly719Asp	rs137852655	Short stature; abnormal bones (Skeletal)	Pseudoachondroplasia
*CTCF*	NM_006565.	c.1670_1674del	p.Cys557*	rs886041901	DD; ID; microcephaly; short stature (Neurological)	Mental retardation type 21
*GAA*	NM_000152.3	c.266G>A	p.R89H	rs200586324	DD; ID; microcephaly; lactic acidosis (Multiple systems)	Pompe disease
*PTPN11*	NM_002834.3	c.417G>C	p.Glu139Asp	rs397507520	Short stature; heart malformation (Multiple systems)	Noonan syndrome
*EDAR^§^*	NM_022336.3	c.486del	p.Ser163Argfs	Gnomad	hydrocephalus, DD; ID; abnormal gait; coarse face; malformation of heart and great vessels; short stature; undescended testis; micropenis (Multiple systems)	
*TTN*	NM_001267550.2	c.36040A>T	p.Lys12014*	Novel	delayed motor development; muscular hypotonia; lactic acidosis (Neurological)	LGMD type 2J
*TTN*	NM_001267550.2	c.68529del	p.Pro22844Leufs	Novel		
*ABCD4*	NM_001353592.1	c.362G>A	p.Arg121His	rs201744101	DD; hypotonia; respiratory distress; aciduria (Neurological)	Methylmalonic aciduria with homocystinuria
*ABCD4*	NM_001353592.1	c.1520C>A	p.A507E	Novel		
*ALPL*	NM_000478.4	c.668G>A	p.R223Q	rs199665722	Short stature; bowed legs; abnormal gait (Skeletal)	Hypophosphatasia
*ALPL*	*NM_000478.4*	*c*.*449 T>G*	*p.I150S*	*Novel*		
*TBK1*	NM_013254.3	c.2079_2082de	p.Glu695Argfs*16	Novel	Juvenile arthritis; abnormal gait; Regression (Neurological)	Amyotrophic lateral sclerosis
*CBL*	NM_005188.3	c.2629G>A	p.Ala877Thr	rs1477997244	DD; vertebral malformations; tracheoesophageal fistula (Multiple systems)	Noonan like syndrome
*LDLR*	NM_000527.4	c.718G>A	p.Glu240Lys	rs137943601		Hypercholesterolemia
*SPINK5*	*NM_001127698.1*	*c.2423C>T*	*p.Thr808Ile*	*rs1212676320*	Congenital ichthyosis (Dermatological)	Netherton syndrome
*ATM*	NM_000051.3	c.7630–2A>C		rs587779866	Ataxia; leukemia (Neurological)	Ataxia‐Telangiectasia
*JAK2*	*NM_004972.3*	*c.1597A>T*	*p.N533Y*	*Novel*		
*LAMA3^#^*	*NM_198129.2*	*c.6115C>T*	*p.(Arg2039Cys)*	*rs138451075*	Difficulty walking; abnormal lower motor neuron morphology (Neurological)	
Homozygous variants						
*LAMA2*	NM_000426.3	c.8244+3_8244+6del		Novel	MD; elevated CK (Neurological)	Congenital muscular dystrophy, merosin‐deficient
*COL4A4*	NM_000092.4	c.1802del	p.P601Qfs	Novel	Nephrotic syndrome; hematuria; progressive hearing loss (Multiple system)	Alport syndrome
*UNC80*	NM_032504.1	c.7697A>C	p.(Glu2566Ala)	Novel	DD; ID; hypotonia, dysmorphic facial features; failure to thrive (Neurological)	NALCN channelopathies
*SZT2*	NM_015284.3	c.7341–2A>G		Novel	DD; Seizures (Neurological)	Epileptic encephalopathy type 18
*MCCC2*	NM_022132.4	c.158 T>C	p.Val53Ala	Novel	DD; ID; hypotonia; failure to thrive; acidosis (Neurological)	3‐Methylcrotonyl‐CoA carboxylase 2 deficiency
*PNPLA1*	NM_001145717.1	c.535C>T	p.Gln179*	Novel	Congenital ichthyosis; keratoderma (Dermatological)	Ichthyosis, congenital, type 10
*NTRK1*	NM_002529.3	c.2205+1G>A		Novel	DD; ID; anhydrosis; insensitivity to pain (Neurological)	Insensitivity to pain, congenital, with anhidrosis
*NALCN*	NM_052867.3	c.3056dup	p.Leu1019Phefs	Novel	DD; ID; hypotonia, regression; dysmorphic facial features (Neurological)	NALCN channelopathies
*PDE6D*	NM_002601.3	c.367_368insG	p.L123Cfs*13	Novel	DD; ID; failure to thrive (Neurological)	Joubert syndrome type 22
*THOC6^§^*	NM_024339.4	c.893del	p.Pro298Gnlfs	Novel	hydrocephalus; DD; ID; abnormal gait; coarse face; malformation of heart and great vessels; short stature; undescended testis; micropenis (Multiple systems)	
*ARSA*	NM_000487.5	c.827C>T	p.Thr276Met	rs74315472	abnormal myelination; developmental regression; hypotonia (Multiple systems)	Metachromatic leukodystrophy
*RNASEH2B*	NM_024570.3	c.529G>A	p.Ala177Thr	rs75184679	Neurological regression (Neurological)	Aicardi‐Goutieres syndrome 2
*PSAT1*	NM_058179.3	c.296C>T	p.Ala99Val	rs587777778	Microcephaly; coarse face; early death (Multiple systems)	Neu‐Laxova type 2
*ALDOB*	NM_000035.3	c.524C>A	p.Ala175Asp	rs76917243	Failure to thrive; lactic acidosis; gastrointestinal problems (Neurological)	Fructose intolerance
*CD27*	NM_001242.4	c.158G>A	p.C53Y	rs397514667	Hepatosplenomegaly; abnormal immune system (Immune/hematology)	Lymphoproliferative syndrome type 2
*PEX7*	NM_000288.3	c.875T>A	p.L292X	rs1805137	DD; short stature; bone malformation; failure to thrive (Neurological)	Rhizomelic chondrodysplasia punctata, type 1
*AP4S1*	NM_007077.4	c.138+3_138+6del		rs876661295	ID; spastic paraplegia (Neurological)	Spastic paraplegia 52
*GBE1*	NM_000158.3	c.986A>G	p.Tyr329Cys	rs80338671	Failure to thrive; hepatosplenomegaly; muscle weakness (Neurological)	Glycogen storage disease type 4
*PLA2G6*	NM_003560.3	c.2370T>G	p.Tyr790*	rs121908680	DD; ID; seizures; cerebellar atrophy (Neurological)	Infantile neuroaxonal dystrophy 1
*EIF2B5*	NM_003907.2	c.407G>A	p.Arg136His	rs958193703	Macrocephaly; ataxia; seizures; regression; leukoencephalopathy; cerebral cysts (Neurological)	Leukoencephalopathy with vanishing white matter
*ARSA*	NM_000487.5	c.433C>G	p.R145G	rs199476373	Abnormal myelination; developmental regression; hypotonia; hyperreflexia; nystagmus (Neurology)	Metachromatic leukodystrophy
*CLN8*	NM_018941.3	c.610C>T	p.Arg204Cys	rs104894060	DD; ID; seizures; regression; cerebellar atrophy (Neurological)	Ceroid lipofuscinosis type 8
*PCCA*	NM_000282.3	c.1209+3A>G		GNOMAD	DD; ID; regression; muscular hypotonia; neuropathy; abnormal hair; failure to thrive; severe encephalopathy; Nerves disease (Neurological)	Propionic acidemia
*FKRP*	NM_024301.4	c.823C>T	p.Arg275Cys	rs1247934219	MD; elevated CK (Neurological)	LGMD type 5C
*CNTNAP1*	NM_003632.2	c.3361C>T	p.Arg1121*	rs142756549	Arthrogryposis; failure to thrive (Neurological)	Lethal congenital contracture syndrome 7
*TBK1^#^*	NM_013254.3	c.2079_2082del	p.Glu695Argfs*16	Novel	Abnormal gait; motor neuron disease (Neurological)	TBK−1 related phenotype
*TWNK*	NM_021830.4	c.1003C>A	p.Pro335Thr	Novel	ID; muscle weakness; seizures; decreased mitochondrial respiratory chain complex activity (Neurological)	Mitochondrial DNA depletion syndrome type 7
*ASPA*	NM_000049.2	c.497C>T	p.Thr166Ile		ID; DD; macrocephaly; regression (Neurological)	Canavan Disease
*VAMP1*	NM_014331.3	c.97C>T	p.Arg33	rs1308616721	Arthrogryposis; myopathic process (Neurological)	VAMP−1 related disorder
*EXT2*	NM_000401.3	c.110C>T	p.Ser37Leu	rs527624522	DD;ID; seizures; microcephaly; failure to thrive (Neurological)	Autosomal recessive EXT2 related syndrome
Hemizygous variants						
*HDAC8*	NM_018486.2	c.562G>A	p.Ala188Thr	Novel	DD; ID; dysmorphic features; microcephaly (Multiple systems)	Cornelia de Lange type 5
*ABCD1*	NM_000033.3	c.1813C>G	p.Leu605Arg	Novel	Neuroregression; hearing problems; adrenal insufficiency (Neurology)	Adrenoleukodystrophy
*HDAC8*	NM_018486.2	c.958G>A	p.Gly320Arg	rs398122909	DD; ID; hirsutism; short stature; microcephaly (Multiple systems)	Cornelia de Lange type 5
*MECP2*	NM_001110792.1	c.509C>T	p.T170M	rs28934906	DD; regression (Neurological)	Rett syndrome
*OPHN1*	NM_002547.2	c.4G>C	p.Gly2Arg	rs1200813419	ID; cerebellar hypoplasia (Neurological)	X‐linked mental retardation with cerebellar hypoplasia

Notes

Only patients with at least one Class 1–2 variant are shown. Variants in italics are class 3. Rows marked with ^§^ represents a patient with pathogenic mutations in three genes. Rows marked with ^#^ represent a patient with pathogenic mutations in two different genes. Variants within the same box were identified in the same patient. The primary pathology identified in the patients is written within parentheses in the “Primary Manifestation” column.

DD: developmental delay; ID: Intellectual disability; CK: creatine kinase; MD: muscular dystrophy.

**Figure 1:**
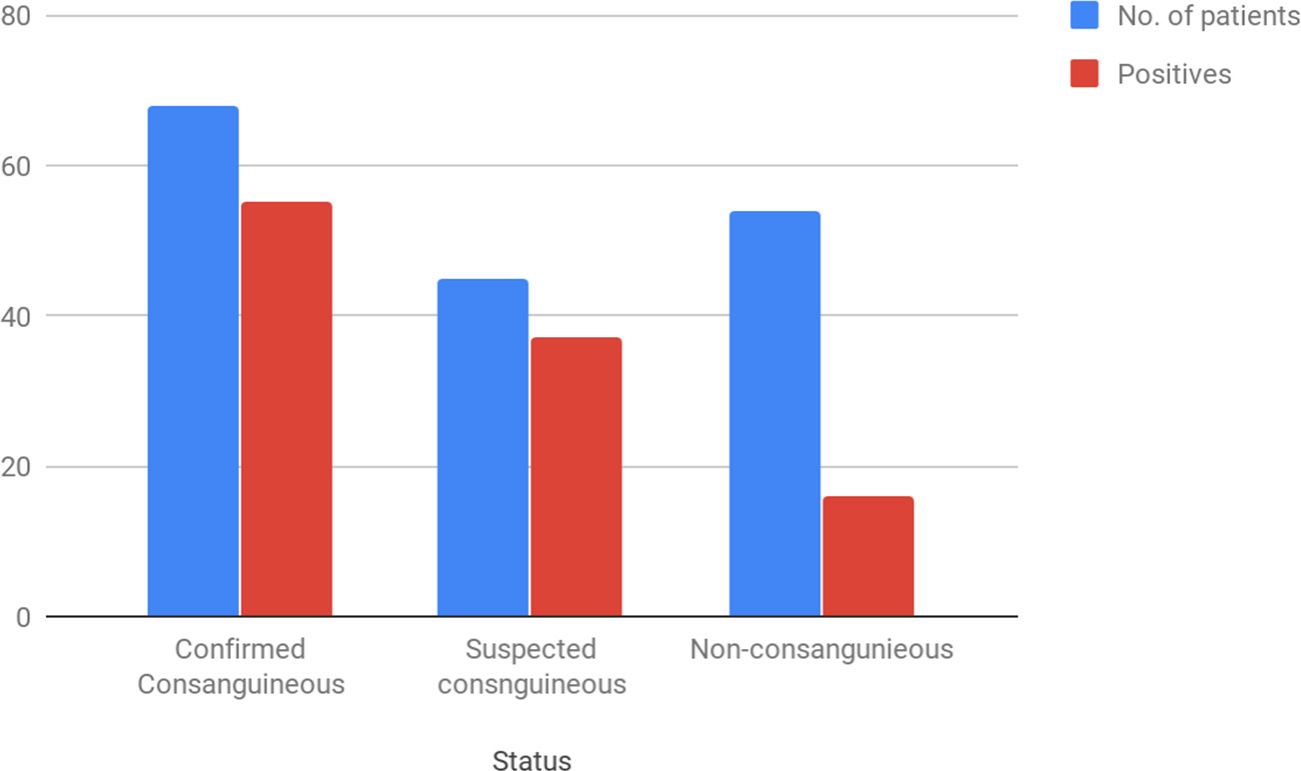
Proportion of WES positives among patients with consanguineous and non‐consanguineous parents

For the patients for whom a WES was performed, 68 (40.7%) had related parents (first degree or second degree cousins). For 45 patients (26.9%), consanguinity was denied although the parents originated from the same village. In consanguineous patients, 55 had a positive result (80.9%). In the 45 patients with parents originated from the same village, 37 (82.2%) had positive results. In patients with non‐related parents (54), 16 had positive results (29.4%) (Figure 1).

**Figure 2:**
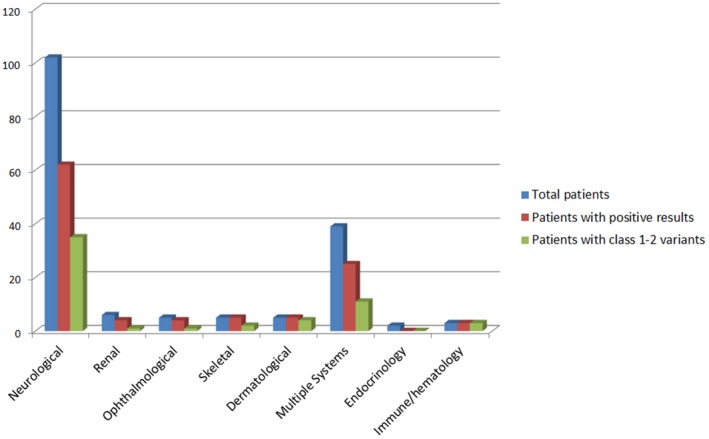
All patients studied by WES categorized according to their primary manifestation

Neurological disorders were the most frequent referral demand for WES: 102/167. Among those, 62 patients had positive results, 35 of them with class 1–2 variants. Five patients with dermatological features and five with skeletal features were referred, and all had positive results, of which four (dermatological) and two (skeletal) had class 1–2 variants. For ophthalmological cases, out of the five referred patients, four had positive results (1 had a class 1–2 variant) and for the renal cases, four out of six were positive, with one patient having one class 1–2 variant. All three patients referred for immune and hematology disease had class 1–2 variants, while the two patients referred for unknown fever and endocrinology had negative results. Finally, 39 patients were referred for multiple anomalies involving many systems. A positive result was noted in 25 of them, where 11 patients had class 1–2 variants (Figure 2).

Ninety‐six percent of the patients/parents of patients (161/167) who underwent WES agreed to receive pathogenic/likely pathogenic variants that were not directly related to their phenotypic features. In 6 cases (3.7%), a positive incidental result was noted.

**Figure 3:**
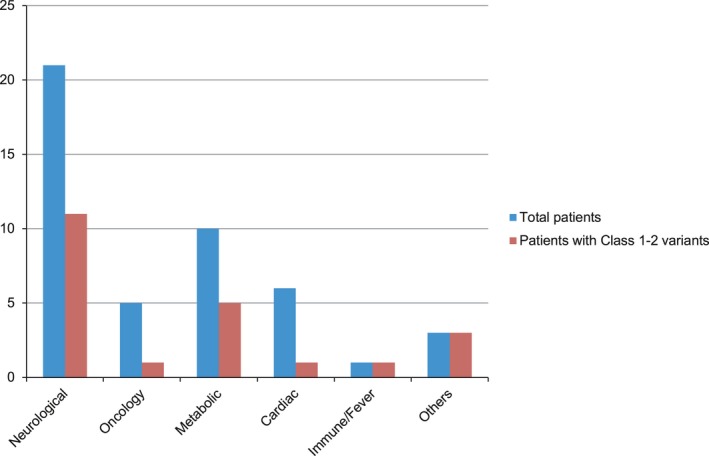
Type of panel employed and positive cases identified

Forty‐six patients, of whom 50.9% came from consanguineous families, underwent multigene panel tests. In 40 (86.9%) of these patients a positive result was found, and 44 variants were identified. In 22 of those patients, 24 class 1–2 variants were found, seven of which were found to be novel (Table 2). A class 1–2 variant was found in 52.3% of the patients that were investigated with a neurological panel; in 20% of the ones with an oncology panel, in 50% of the metabolic panel, 16.6% of cardiac panel, and 100% of the renal, dermatological and primary immunodeficiency and fever of unknown origin panels (Figure 3). Six patients (13%) had negative results. None of the patients with negative results had any deletion/duplication detected following MLPA analysis.

**Table 2:** Variants identified by multigene panel testing

Gene	Transcript	cDNA	Protein	Novelty	Primary manifestation	Diagnosis post panel
Heterozygous variants						
LAMA2	NM_001079823.1	c.3829C>T	p.Arg1277*	Novel		
LAMA2	NM_001079823.1	c.1300C>T	p.Arg434*	rs1374568851	MD; elevated CK (Neurological)	Merosin deficiency
CFTR	NM_000492.3	c.3846G>A	p.Trp1282*	rs77010898	Chronic pancreatitis (Metabolic)	Chronic pancreatitis
CFTR	NM_000492.3	c.3883_3886del	p.Ile295Phefs	Novel		
CFTR	NM_000492.3	c.3909C>G	p.Asn1303Lys	rs80034486	Chronic pancreatitis (Metabolic)	Chronic pancreatitis
*CFTR*	*NM_000492.3*	*c.1211G>T*	*p.Gly404Val*	*rs1324302547*		
SCN1A	NM_001165963.2	c.4907G>A	p.Arg1636Gln	rs121917995	Early seizures (Neurological)	Dravet syndrome
SCN1A	NM_001165963.2	c.2593C>T	p.Arg865*	rs794726697	Early seizures (Neurological)	Dravet syndrome
TSC2	NM_001114382.2	c.1832G>A	p.Arg611Gln	rs28934872	ID; DD; seizures (Neurological)	Bourneville Tuberous sclerosis
NF1	NM_001128147.2	c.499_502delTGTT	p.Cys167Glnfs	rs786201874	Café‐au‐lait spots (Dermatological)	Neurofibromatosis
RB1	NM_000321.2	c.2247_2248insAA	p.Asp750Lysfs	Novel	Bilateral Retinoblastoma (Oncological)	Retinoblastoma
FBN1	NM_000138.4	c.7713T>G	p.Cys2571Trp	Novel	Tall stature (Neurological)	Marfan syndrome
SOS1	NM_005633.3	c.1352C>A	p.T451K	rs730880218	ID; DD; cardiac malformation (Cardiac)	Noonan syndrome
WT1	NM_024426.4	c.1250G>T	p.Gly417Val	rs869025561	Nephrotic syndrome (Renal)	Nephrotic syndrome type 4
Homozygous variants						
MMACHC	NM_015506.2	c.271dup	p.Arg91Lysfs	rs398124292	ID; DD; failure to thrive (Metabolic)	Methylmalonic aciduria
BCKDHB	NM_183050.3	c.995C>T	p.Pro332Leu	Novel	Ketosis; lactic acidosis; elevated leucine‐isoleucine‐valine (Metabolic)	Maple syrup urine disease
GALNS	NM_000512.4	c.898+1G>A		rs761850746	Short stature; severe scoliosis (Neurological)	Mucopolysaccharidosis type IVA
SGCG	NM_000231.2	Deletion of exon 7		Reported	MD; slightly elevated CK (Neurological)	Limb‐girdle muscular dystrophy type 2C
MMACHC	NM_015506.2	c.472T>C	p.Phe158Leu	rs201312386	Ketosis; lactic acidosis; elevated leucine‐isoleucine‐valine (Metabolic)	Maple syrup urine disease
JAK3	NM_000215.3	c.2141C>T	p.Thr714Met	rs140655992	Failure to thrive; recurrent infections; diarrhea (Immune/fever)	Severe combined immunodeficiency
LAMA3	NM_198129.2	c.1789–7_1789–5delTTC		Novel	Epidermolysis bullosa (Dermatological)	Epidermolysis bullosa Herlitz type
Hemizygous variants						
DMD	NM_004006.2	c.4071+1G>A		rs1060502643	MD; elevated CK (Neurological)	Duchenne muscular dystrophy
DMD	NM_004006.2	c.1283del	p.Asn428Ilefs	Novel	MD; elevated CK (Neurological)	Duchenne muscular dystrophy
SLC6A8	NM_005629.3	c.1661C>T	p.Pro554Leu	rs397515559	ID; DD; failure to thrive; microcephaly; seizures (Neurological)	Cerebral creatine deficiency syndrome
GJB1	NM_001097642.2	c.164_184dup	p.Thr55_An61dup	Novel	Muscle atrophy; gait disturbance; reduced motor nerve conduction (Neurological)	Charcot‐Marie‐Tooth neuropathy X type 1

Only patients with at least one Class 1–2 variant are shown. The variant in italic is a class 3 variant. Variants within the same box were identified in the same patient. The type of panel used is mentioned within parentheses under the “Primary Manifestation” column.

DD: developmental delay; ID: Intellectual disability; CK: creatine phosphokinase; MD: muscular dystrophy.

## 4 DISCUSSION

We applied WES and multigene panels for molecular diagnosis in 213 pediatric patients referred from different areas across Lebanon. Out of all patients combined, a pathogenic or likely pathogenic variant leading to a molecular diagnosis was found in 79 patients (37.1%). This diagnostic rate was 34.1% for patients studied by WES and 47.8% for those analyzed by multigene panels. The higher diagnostic rate for panels was expected, since panels were ordered mainly in the cases where the clinician was relatively more confident about characterizing the underlying genetic condition. Recent studies have noted higher diagnostic yields from WES in pediatric cohorts with suspected monogenic disorders (Charng et al., 2016; Dillon et al., 2018; Tan et al., 2017). This could be due to the fact that physicians in Lebanon tend to order WES analysis only for complex cases, and rarely when they have a relatively strong clinical suspicion to help them along. It is important to note that in some cases (about 10%–15% of referrals), the diagnosis was rightfully suspected, and thus causal mutation could have been identified by Sanger sequencing, however, parents opted for WES or gene panels instead, because of the relatively high cost of Sanger sequencing. Moreover, parents were more inclined to opt for WES rather than panels because of the ability of WES to uncover incidental findings and because the cost of the two does not differ significantly.

**Figure 4:**
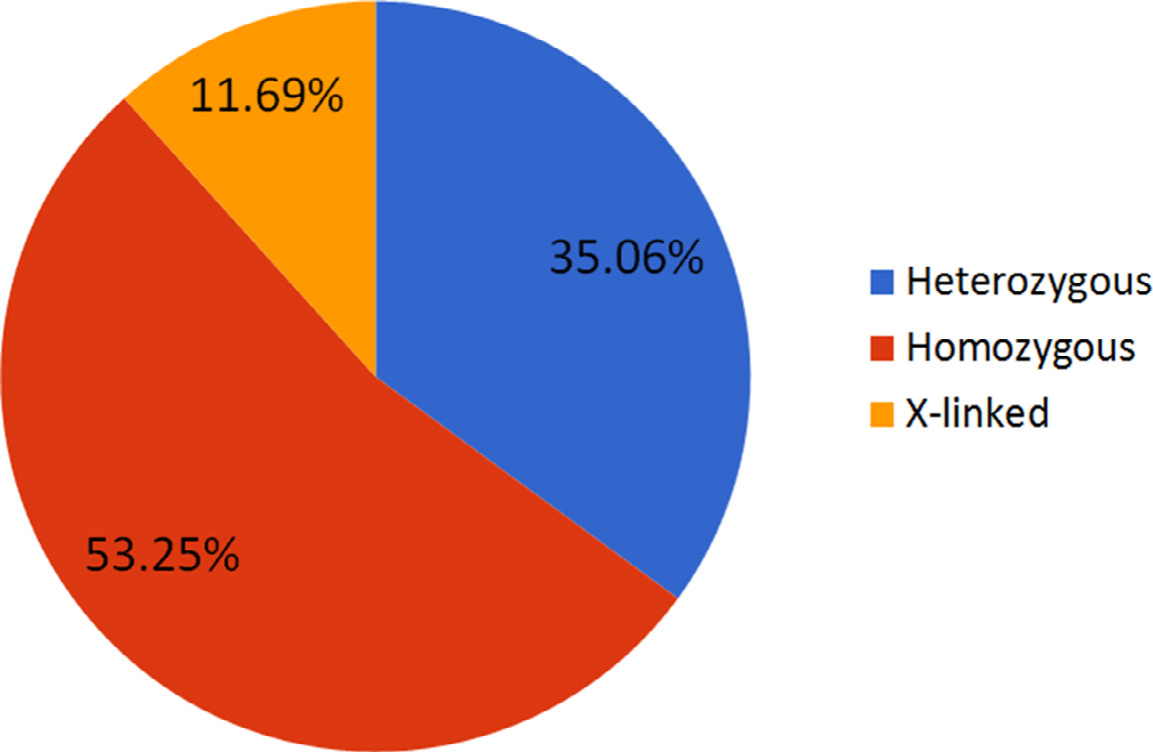
Mode of inheritance in patients identified with class 1 and class 2 variants by both WES and panel studies

Out of the 79 patients who had class 1–2 variants, 53.2% had an autosomal recessive disorder, 35.4% an autosomal dominant disorder, and 11.4% a X‐linked disorder (Figure 4). The relatively high number of autosomal recessive disorders is most probably secondary to the high consanguinity rate. This is consistent with several previous reports from the Arab region in areas that exhibit high rates of consanguinity (Alfares et al., 2017; Al‐Shamsi, Hertecant, Souid, & Al‐Jasmi, 2016). Indeed, in total, nearly 60% of the patients had related parents or are suspected to have related parents. The percentage of positive cases in the families which denied consanguinity but were from the same village (82.2%) is comparable to that in consanguineous families (80.9%), suggesting that the in the former, the parents could indeed be related (Figure 1).

Neurological problems were the most frequent referral demand for both WES and multigene panels (122/213)**.** It is noteworthy that in the neurological referral demands for WES, seven of the patients were autistic, and in none of them a positive result in any relevant gene(s) was found, suggesting that NGS in purely autistic patients may offer little benefit.

In some cases, genetic testing finds that the patient has more than one variant involved in the pathogenicity of the disease (Lupski, Belmont, Boerwinkle, & Gibbs, 2011; Megarbane, 2018). In such instances, several rare variants are shown to cause a disease in combination, one being the “Highly penetrant Mendelizing Variant”, responsible for the disease, and other variant(s) modifying the phenotype (Lupski et al., 2011). As the pipeline we followed for identifying causal variants relied on checking for variants in genes already known to be associated with relevant phenotypes, our study was therefore not suited to easily diagnose the cases where the association of variants in different genes could be at the origin of the disease. For instance, in this study, only seven patients (six from the WES series and one from the panel series) had more than one variant detected, one of which was classified as pathogenic or likely pathogenic, and the other was a class 3 variant (variant of unknown significance). The class 3 variants in all six of the WES patients were in genes different from the primary gene carrying the Class 1 or 2 mutation. Additionally, one patient was identified to carry pathogenic mutations in three different genes (Tables 1 and 2).

In three consanguineous families, we observed autosomal recessive inheritance of genes previously reported as showing dominant inheritance patterns, namely *VAMP1* (OMIM 185,880)*, TBK1* (OMIM 604,834) and *EXT2* (OMIM 608,210) genes. In those three families, parents were heterozygous and healthy. Similar observations have been previously reported in the Arab region (El Bazzal, Atkinson, Gillart, Delague, & Mégarbané, 2018; Monies et al., 2017). This further emphasizes the importance of not dismissing variants that do not fit previously reported patterns of inheritance (Monies et al., 2017).

In nearly a third of the patients, a potential positive result was obtained as only a class 3 variant was found. In three of these cases, the possibility to study the segregation of the disease allowed us to rule out the involvement of the class 3 variant in the pathology.

Certain findings within our study highlight the importance of accompanying NGS analysis with an informed and specialized interpretation by a geneticist, coupled with proper genetic counseling. For instance, one patient with a class 2 mutation was re‐evaluated and classified as a class 5 variant. Three patients had false negative results. For those, a causal mutation was found after Sanger sequencing of the suspected genes (these variants were not listed in Table 1 as they were not identified by WES). Reviewing the fastq files showed that the involved genes were not fully covered. In another example, in two patients with recessive conditions, WES was able to identify only single heterozygous variations in relevant genes. However, because of the strong phenotype‐genotype correlation in these cases, we further studied the respective genes by Sanger sequencing. In the first of these cases, this approach enabled the identification of a second pathogenic variant in the *ABCD4* gene (OMIM 603,214). However, in the second case, a patient with a heterozygous variant in the *ATM* gene (OMIM 607,585), we were unable to find a second mutation even after an MLPA exam. RNA analysis will be performed soon.

Moreover, around 65 of the 213 cases (30.5%) remain unsolved as of the time of writing. As most of the cases tested in WES were solo cases, and because of the pipeline used for analyzing the results, we were unable to find any novel candidate genes in our patients which we believe could explain the origin of the pathology in many cases as it was showed in other reports (Monies et al., 2017). With this in mind, in six families with more than two affected sibs further investigations are pending. Furthermore, negative results could in part be due to insufficient coverage or alternatively because, for some patients, a large number of variants were identified, making it difficult to pinpoint the causative variant without any segregation analysis. It is worth noting that for three patients who had negative results, an array CGH was performed and a pathogenic variation was found in one.

Three couples who presented with a history of prior affected children were offered duo tests because none of the affected children were available for testing. For these families, we were able to identify the likely causal mutation, however they were not included in this paper.

In 96% of cases where WES was performed, the patients or their parents agreed to receive any incidental findings classified as pathogenic/likely pathogenic, even if they are not related to the original referral phenotype. Our study identified six patients who had incidental class 1 or class 2 mutations in genes belonging to the 59 actionable genes as recommended by ACMG (Kalia et al., 2017). In one of these patients, the incidental finding was related to a risk of sudden death. A familial screening was performed and the carrier members were referred to cardiac specialist for better follow‐up. This result emphasizes the importance of genetic counselling, which unfortunately lacks strongly in Lebanon (Nakouzi et al., 2015).

As is the case in many developing countries, the implementation and wide adoption of NGS has been hindered mostly by the costly finances associated with establishing and running a sequencing facility as well as the lack of expertise, and the cost of such genetic services (Helmy, Awad, & Mosa, 2016). Lebanon suffers from a scarcity of clinical geneticists and a lack of genetic counseling services (Nakouzi et al., 2015). This is especially a problem, given the high number of genetic disorders in the Lebanese population (Nakouzi et al., 2015) and the sudden increase in the number of residents in Lebanon, given the huge influx of Syrian refugees that Lebanon has witnessed in the recent few years (UNHCR, 2017). These Syrian refugees, in addition to the Palestinian (UNHCR, 2016) and Iraqi (UNHCR, 2017) refugees are considered a burden on the health sector, and are not granted any government health coverage (Santoro & McKee, 2017). For most of these refugees, as well as a lot of Lebanese citizens, genetic testing has to be personally financed, as they are not covered by national health insurance (social security) neither private insurance companies. The financial cost that a patient's family has to incur has thus made a lot of families opt out of the recommended genetic testing.

Our study showed 69.5% positive results for WES and panels combined, emphasizing the utility and diagnostic power of NGS techniques. The latter has helped to obtain a diagnosis more rapidly and more accurately, potentially allowing for a more efficient genetic counseling. It also reduced the number of unnecessary and costly laboratory tests. This thus highlights the importance of improving the adoption of such techniques in Lebanon as well as enabling access of citizens as well as temporary residents to NGS tools.

## CONFLICT OF INTEREST

The authors have no conflict of interest to declare.

## AUTHOR CONTRIBUTION

PN, SS, HM, AF, GH, PN, RD, HM, SH, SA, ML, RF, DH, MR, SS, OT, PAJ, GC, RS, ACG, MAA, SEH, VD, AM have made substantial contributions to conception and design and for important intellectual content. PN, SH, SS, ACG, MAA, SEH, VD, AM have made substantial contributions in acquisition of data, analysis and interpretation of data. PN, SEH, VD, AM, have been involved in drafting the manuscript and revising it critically. All authors have given final approval of the version to be published.
